# Prospective multicenter non-interventional real-world study to assess the patterns of use, effectiveness and safety of follitropin delta in routine clinical practice (the PROFILE study)

**DOI:** 10.3389/fendo.2022.992677

**Published:** 2022-12-22

**Authors:** Christophe Blockeel, Georg Griesinger, Rocco Rago, Per Larsson, Yum Lina Yip Sonderegger, Stéphane Rivière, Joop S. E. Laven

**Affiliations:** ^1^ Centre for Reproductive Medicine, Universitair Ziekenhuis Brussel, Vrije Universiteit Brussel, Brussels, Belgium; ^2^ Department of Gynecological Endocrinology and Reproductive Medicine, University Hospital of Schleswig-Holstein, Luebeck, Germany; ^3^ Physiopathology of Reproduction and Andrology Unit, Department of Gender, Parenting, Child and Adolescent Medicine, Sandro Pertini Hospital, Rome, Italy; ^4^ Global Biometrics, Global Clinical Development, Ferring Pharmaceuticals, Copenhagen, Denmark; ^5^ Ferring Pharmaceuticals, Ferring International Center SA, Saint-Prex, Switzerland; ^6^ Division of Reproductive Endocrinology and Infertility, Department of Obstetrics and Gynaecology, University Medical Center, Rotterdam, Netherlands

**Keywords:** ovarian stimulation, individualized algorithm-based dosing, follitropin delta, real-world evidence, pregnancy

## Abstract

**Objective:**

To observe the real-world utilization patterns, effectiveness and safety profile of follitropin delta in women ≥18 years naïve to ovarian stimulation undergoing *in vitro* fertilization (IVF) or intracytoplasmic sperm injection (ICSI).

**Design:**

Prospective, multinational, multicenter, observational study. All IVF/ICSI treatment protocols were conducted according to routine clinical practice, including undertaking fresh/frozen transfers. Outcomes included use of dosing algorithm, follitropin delta dosing patterns, ovarian response, pregnancy rates and adverse drug reactions (ADRs).

**Results:**

The first ovarian stimulation cycle using follitropin delta was initiated in 944 women. Mean baseline demographics were: age, 33.5 ± 4.7 years; bodyweight, 67.1 ± 13.6 kg; anti-Müllerian hormone, 20.3 ± 16.1 pmol/L (2.84 ± 2.25 ng/mL). The dosing algorithm was used to calculate the follitropin delta daily starting dose in 893/944 women (94.5%). The mean difference between the calculated and prescribed daily dose was small (0.2 ± 1.40 µg). The mean daily starting follitropin delta dose was 10.4 ± 2.72 µg and the mean total dose administered was 104 µg. Follitropin delta dose adjustments were reported for 57/944 (6.0%) women. The mean number of retrieved oocytes was 10.1 ± 7.03. Ongoing pregnancy at 10–11 weeks was reported for 255 women (27.0% per initiated cycle and 43.1% per fresh transfer [n=592]). Cumulative ongoing pregnancy rate after fresh and/or frozen transfer was 36.4% (344/944). Four women discontinued follitropin delta due to ADRs. Ovarian hyperstimulation syndrome (OHSS) was the most frequently reported ADR (n=37 [3.9%]); most cases of OHSS were of mild or moderate intensity (n=30 [3.2%]).

**Conclusions:**

This large real-world study of follitropin delta utilization patterns confirms its good pregnancy rates while minimizing OHSS risk during first ovarian stimulation cycle.

## 1 Introduction

Success of *in vitro* fertilization (IVF) and intracytoplasmic sperm injection (ICSI) techniques used to help couples conceive depends on obtaining enough oocytes to create high-quality embryos for transfer while minimizing the risk of ovarian hyperstimulation syndrome (OHSS). Physicians use different parameters to predict ovarian response, including anti-Müllerian hormone (AMH) levels or antral follicle count (1–3). In clinical practice, treatment individualization is not always based on evidence but rather the physician’s experience (4). Follitropin delta (REKOVELLE^®^, Ferring Pharmaceuticals, Switzerland), is the first recombinant human follicle-stimulating hormone (FSH) to be produced in a human cell line and is also the first FSH that has an approved algorithm based on the woman bodyweight and baseline serum AMH to individualize dosing (5, 6). The dosing algorithm was developed to reduce the risk of extreme hypo- and hyper-ovarian response while maintaining ongoing pregnancy rates compared with conventional FSH dosing strategies (5, 7–14).

Various follitropin preparations are commercially available, including naturally occurring highly purified urofollitropin, recombinant follitropins that are produced using cultured cell lines, and biosimilars (15). Different follitropins share the same amino acid sequence and tertiary protein structure but vary in their post-translational modifications (glycosidic complexity, sialylation and sulfation patterns), which affect *in vivo* bioactivity (16). Follitropin delta’s post-translational modifications closely resemble the glycosylation profile of endogenous human FSH, more than recombinant follitropins alfa and beta which are derived from Chinese hamster ovary cell lines (16).

The efficacy and safety of individualized dosing of follitropin delta compared with standard dosing of 150 IU daily recombinant FSH has been established in randomized controlled trials (RCTs). The Phase 3 ESTHER-1 trial demonstrated follitropin delta to be an efficacious and well-tolerated treatment for ovarian stimulation (OS), with a reduced risk of OHSS and a reduced need for gonadotropin-releasing hormone (GnRH) agonist as a preventive intervention for OHSS compared with follitropin alfa (11, 17). Follitropin delta has also demonstrated low immunogenicity potential with second or third repeated OS in the ESTHER-2 trial (18). The Phase 3 STORK trial in Japanese women undergoing IVF/ICSI established non-inferiority between follitropin delta and follitropin beta based on number of oocytes retrieved as well as a favorable benefit-risk with follitropin delta (12). The Phase 3 GRAPE trial established non-inferiority for ongoing pregnancy rates with follitropin delta dosing versus follitropin alfa in Asian women, as well as a significantly higher live birth rate and significantly fewer early OHSS and/or preventive interventions compared to follitropin alfa (13).

Despite this evidence, translating results from RCTs into a clinical setting can be challenging because interventional trials are performed according to stringent treatment protocols, with randomized treatment allocation to reduce risk of bias and strict eligibility criteria to reduce confounding variables. As such, many patients or treatment conditions that are found in daily clinical practice are not always represented in interventional trials. Moreover, some trials investigating drugs for OS may have protocol-driven treatment pathways and pre-determined decision points that affect, or relate to, specific outcomes (19, 20). Protocols for OS are also hugely variable (21). The aim of our single arm, no comparator, observational study, PROFILE, is to report real-world treatment patterns of follitropin delta, including the use of the individualized dosing algorithm, as well as effectiveness and safety profile, in a broad range of women naïve to IVF and ICSI undergoing up to three cycles with follitropin delta.

## 2 Materials and methods

### 2.1 Study design

PROFILE was a prospective, multicenter real-world, observational study in women who had not previously undergone IVF/ICSI treatment. The study was performed in compliance with the Declaration of Helsinki, current Guidelines for Good Pharmacoepidemiology Practice and other national laws applicable in the countries where the study took place, including local institutional review board ethics approval. All women provided written informed consent as part of the enrolment process. Women were enrolled only after the decision to treat with follitropin delta had been made. No aspect of this study interfered with or influenced routine clinical procedures, or the medications prescribed to participating women. All data were collected as part of routine clinical practice at each study site. No study drugs were reimbursed or provided by the study sponsor. Each woman could continue in the study for a maximum of three treatment cycles. The ClinicalTrials.gov identifier is NCT03393780.

### 2.2 Study participants

In countries where follitropin delta had marketing approval and was available at specialist reproduction medicine clinics, women prescribed follitropin delta for their first IVF/ICSI treatment were consecutively invited to participate in the study. Women who were ≥18 years, IVF/ICSI treatment-naïve and scheduled for OS with follitropin delta for their first cycle of IVF/ICSI using fresh or frozen ejaculated sperm from a male partner or sperm donor were eligible for inclusion. Women who were already participating in an ongoing interventional clinical trial which required any treatment or follow up were excluded from enrolment. Women with any contraindications for treatment with follitropin delta, and women who were planning to become oocyte donors or undergoing OS for fertility preservation were also excluded from enrolment.

### 2.3 Study drug

As this was a post-authorization, non-interventional observational study, participating physicians could decide all drug doses and regimens for the participating women, including for follitropin delta. All participating physicians were provided with the approved follitropin delta starting dose algorithm (via an on-line tool or App; Ferring Pharmaceuticals, Switzerland), which is based on a woman’s body weight and serum AMH. The approved starting daily dose of follitropin delta for women with AMH <15 pmol/L is 12 µg, irrespective of bodyweight. For women with AMH ≥15 pmol/L the daily dose is decreased from 0.19 to 0.10 µg/kg according to increasing AMH concentration until AMH ≥40 pmol/L (6). Follitropin delta was administered subcutaneously using its pen injection device.

A recent (baseline) AMH measurement was acquired for each participating woman using local laboratory facilities. At the time of the study, the approved AMH assay available to use with the follitropin delta dosing algorithm was the Elecsys^®^ AMH Plus (Roche Diagnostics International, Switzerland) with a measuring range from 0.01 to 23 ng/mL (0.07 to 164 pmol/L), and repeatability and intermediate precision of 1.7–2.6% and 2.1–2.9%, respectively (22).

### 2.4 Outcomes

The primary endpoint was the real-world treatment patterns of follitropin delta, including starting daily dose, number of days of treatment, deviations from the approved dosing schedule as per the summary of product characteristics (per-label), use of dosing algorithm, and use of other treatments during OS, such as GnRH protocol, triggering methods of follicle maturation and luteal phase support.

The key secondary endpoint of ovarian response in Cycle 1 (total number of oocytes retrieved) was documented for all women and for four subgroups based on baseline serum AMH concentration (<7, ≥7 and <15, ≥15 and ≤35 and >35 pmol/L). AMH concentration is a known predictor of ovarian response (1–3). Pregnancy outcomes were recorded, comprising human chorionic gonadotropin [hCG] test, clinical pregnancy defined as at least one gestational sac 5–6 weeks after transfer, vital pregnancy defined as at least one intrauterine gestational sac with fetal heartbeat 5–6 weeks after transfer and ongoing pregnancy (at least one intrauterine viable fetus 10–11 weeks after transfer). Pregnancy data were reported for all women who initiated Cycle 1 by GnRH protocol subgroup (antagonist or agonist), as well as follitropin delta monotherapy and mixed FSH therapy subgroups.

Other secondary endpoints comprised number of women with cycle cancellation for Cycle 1 (including reasons for cancellation) and description of preventive interventions used for potential OHSS for Cycle 1. The number of women with early and late OHSS of severe intensity was recorded for all initiated cycles as well as time from triggering to OHSS occurrence, OHSS duration, duration of hospitalization, medical and surgical interventions, and the OHSS outcomes. Treatment for OHSS and preventive treatment for potential OHSS were conducted according to local practices. All adverse drug reactions (ADRs) and serious ADRs, including OHSS, were reported using the Medical Dictionary for Regulatory Activities (MedDRA) preferred terms (version 23.1). Serious ADRs were defined as an ADR that resulted in death, or was life-threatening, required in-patient hospitalization or prolonged any existing hospitalization, resulted in persistent or significant disability, was a congenital anomaly/birth defect or was an important medical event requiring intervention to prevent any of the previous definitions of a serious ADR.

### 2.5 Statistical analysis and determining sample size

Dosing patterns and effectiveness were assessed for Cycle 1. Safety was reported for all cycles. Descriptive statistics were used for all outcomes. Pregnancy outcomes were reported for all women who initiated ovarian stimulation with follitropin delta in Cycle 1. The rate of ongoing pregnancy and cumulative ongoing pregnancy were also reported per embryo/blastocyst transfer. At the time of study termination, if a participant had a vital pregnancy reported as ‘Yes’ and ongoing pregnancy was missing, the ongoing pregnancy was regarded as ‘Pending’. If the last vital pregnancy was ‘Yes’ and the last ongoing pregnancy result from the same fresh or frozen transfer was missing, the cumulative ongoing pregnancy was regarded as ‘Pending.’

A study sample size of between 1000–1200 participants was calculated based on an adverse drug reaction rate range of 2.5–50%, expected drop-out of 20% and a precision (certainty of results) of 0.9–3.1%.

## 3 Results

### 3.1 Study participants and enrollment

**Figure 1:**
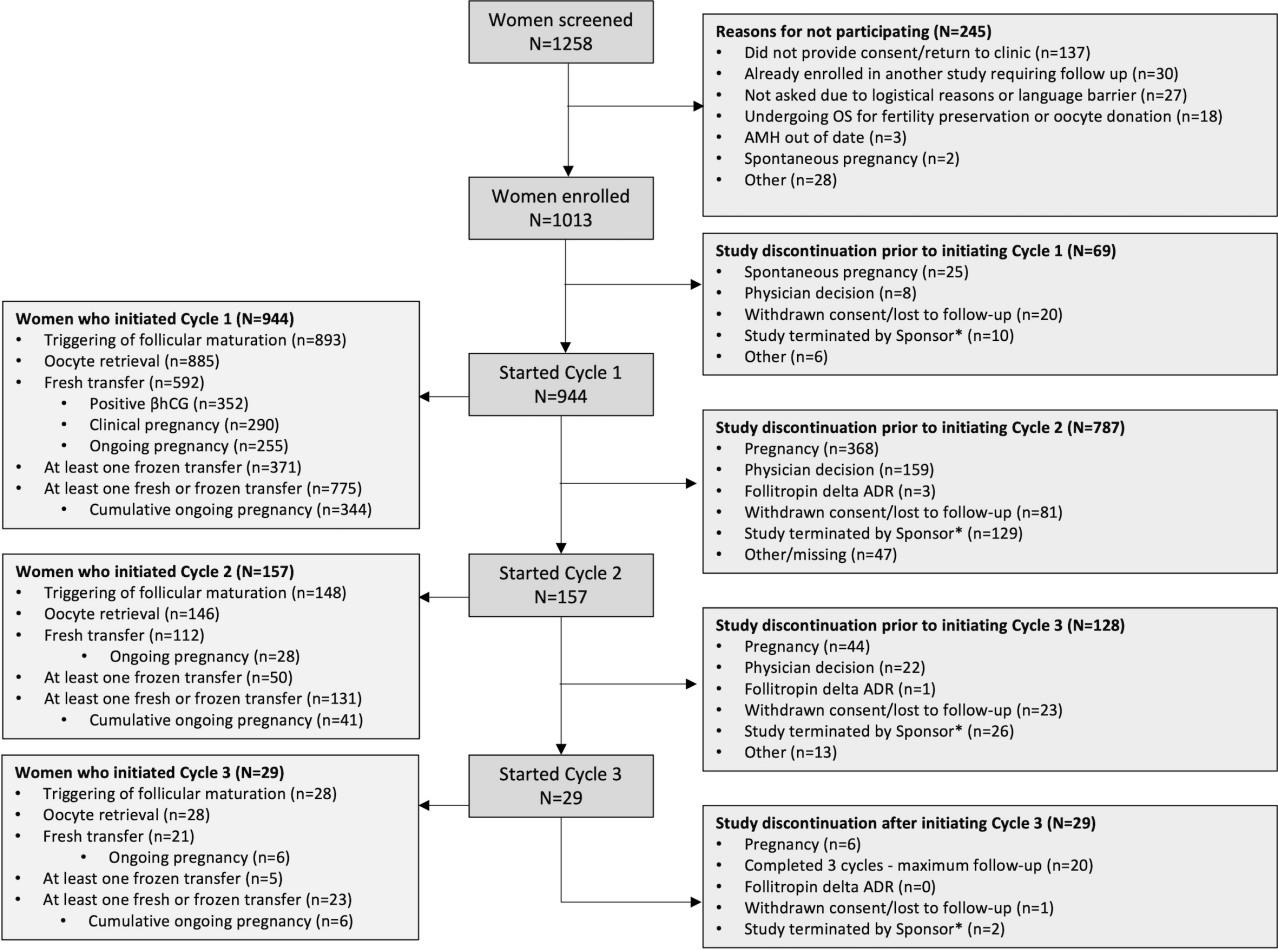
Patient study flow *Patient follow-up was truncated at the global cut-off date of 02 October 2020. ADR, adverse drug reaction; OS, ovarian stimulation.

A total of 1258 women were screened, of whom 1013 (80.5%) met the inclusion and exclusion criteria and were enrolled in the study between March 2018 and October 2020. The study was terminated early by the study sponsor due to the COVID-19 pandemic during which many fertility clinics closed or provided reduced services, but an adequate number of women had enrolled to fulfil the analysis of the primary endpoint. There were 34 study sites across 10 countries. The largest proportion of women was enrolled in Belgium (37.3%), followed by the Netherlands (14.5%), Germany (14.2%) and Italy (7.6%). Patients were also recruited in Australia, Austria, Canada, Poland, Spain and United Kingdom. Of the 245/1258 women who were not enrolled, the most common reason was non-consent to participate, or they did not return to the clinic (n=137 women; **Figure 1**). A total of 69 women discontinued the study prior to starting their first OS cycle and 944 women started their first OS cycle (Cycle 1), of whom 157 and 29 women also initiated Cycle 2 and Cycle 3, respectively. The most common reason for discontinuation prior to Cycle 2 was pregnancy (n=368). Four spontaneous pregnancies were reported during Cycle 1, and another 20 spontaneous pregnancies were reported prior to women commencing Cycle 2.

The 944 women who initiated Cycle 1 had a mean age of 33.5 ± 4.7 years and mean bodyweight of 67.1 ± 13.6 kg (**Table 1**). Their mean baseline AMH concentration was 20.3 ± 16.1 pmol/L (2.84 ± 2.25 ng/mL); 17.6% of women had a low AMH concentration of <7 pmol/L (0.98 ng/mL) and 15.3% had a high AMH of >35 pmol/L (4.9 ng/mL). The main type of infertility was primary infertility reported by 671 women (71.1%) and the mean duration of infertility was 2.7 years. The most common reason for infertility was male factor, which was reported by 411 women (43.5%), followed by unexplained infertility, reported by 227 women (24.0%). The mean menstrual cycle duration was 30.3 days. Women who initiated Cycles 2 and 3 had an overall comparable reproductive history as Cycle 1 (data not shown).

**Table 1:** Demographics and baseline characteristics for Cycle 1.

Patient characteristic	All (N=944)
Age, years	33.5 ± 4.7
Body mass index, kg/m^2^ (n=937)	24.2 ± 4.6
Bodyweight, kg	67.1 ± 13.6
Bodyweight category	
<50 kg	42 (4.4)
≥50 and < 60 kg	256 (27.1)
≥60 and <70 kg	299 (31.7)
≥70 and <80 kg	165 (17.5)
≥80 and <90 kg	108 (11.4)
≥90 and <100 kg	39 (4.1)
≥100 kg	22 (2.3)
Missing	13 (1.4)
Baseline AMH (pmol/L)	20.3 ± 16.1 16.4 (8.8–27.1)
Baseline AMH category, n (%)	
<7 pmol/L	166 (17.6)
≥7 and <15 pmol/L	259 (27.4)
≥15 and ≤35 pmol/L	375 (39.7)
>35 pmol/L	144 (15.3)
Duration of infertility, years	2.7 ± 2.1
Type of infertility	
Primary infertility	671 (71.1)
Secondary infertility	272 (28.8)
Missing	1 (0.1)
Reason(s) for infertility, n (%)*	
Unexplained infertility	227 (24.0)
Tubal infertility	134 (14.2)
Male factor	411 (43.5)
Anovulatory infertility WHO Group I	25 (2.6)
Anovulatory infertility WHO Group II	64 (6.8)
Endometriosis	104 (11.0)
Other	176 (18.6)
Missing	4 (0.4)

Data are mean ± SD, median (range) or n (%).

N, number of patients; SD, standard deviation.

7 pmol/L = 0.98 ng/mL; 15 pmol/L = 2.1 ng/mL; 35 pmol/L = 4.9 ng/mL.

Percentages calculated using total number of women in study (N).

*Percentages sum to >100% because women could be included in more than one category.

### 3.2 Follitropin delta dosing patterns

**Figure 2:**
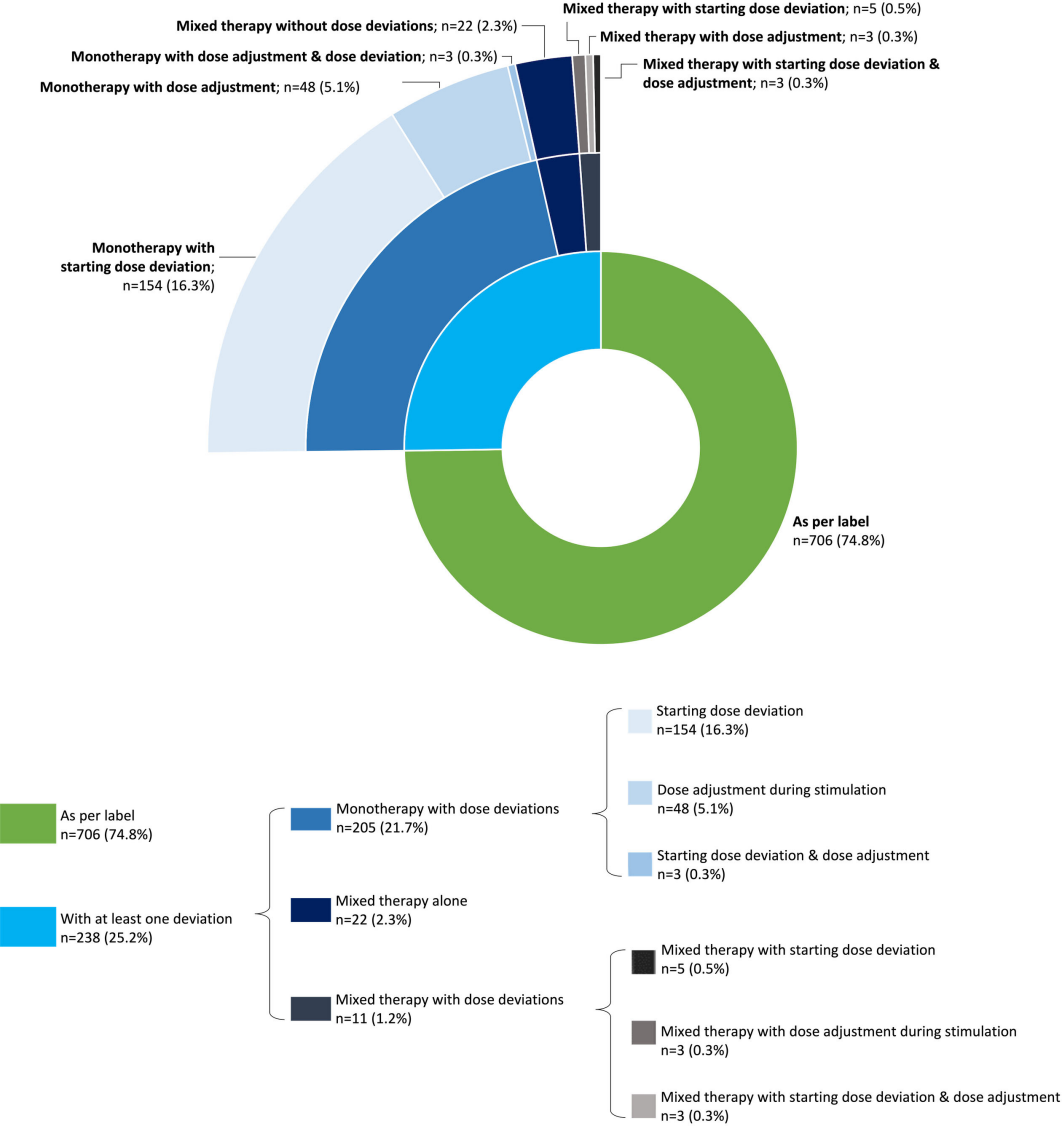
Main deviations with follitropin delta during Cycle 1. The figure depicts the hierarchical distribution of deviations in follitropin delta use at Cycle 1. The inner circle differentiates between women who received follitropin delta stimulation therapy as per label (in green) and the women who had at least one deviation in the expected treatment pattern (in blue). The first outer circle presents the group of women with a regimen deviation (i.e., mixed FSH therapy; in dark blue), a dose deviation (including starting dose deviations and dose adjustments during stimulation; in lighter blue) and women who had both regimen and dose deviations (in grey). The second outer circle presents the distribution according to the type of dose deviation (starting dose deviation or dose adjustment during stimulation). FSH, follicle-stimulating hormone.

In Cycle 1, 706/944 women (74.8%) received follitropin delta following the label indication, i.e., as monotherapy without a starting dose deviation and/or adjustment during stimulation (**Figure 2**).

#### 3.2.1 Overview of starting dose, total dose and duration of treatment for Cycle 1

For the 944 women who initiated Cycle 1, the mean starting dose was 10.4 µg, the mean total dose administered was 104 µg and the mean duration of treatment was 10.0 days (**Table 2**).

**Table 2:** Follitropin delta treatment patterns during Cycle 1.

	Follitropin delta as FSH monotherapy		Follitropin delta as part of combination FSH therapy (N=33)	All(N=944)
	GnRH antagonist(N=827)	GnRH agonist(N=84)	
Overall follitropin delta dosing				
Daily starting dose prescribed (µg)	10.3 ± 2.76	10.8 ± 2.02	11.1 ± 3.12	10.4 ± 2.72
Duration of stimulation (days)	9.9 ± 2.24	10.9 ± 2.06	10.2 ± 2.57	10.0 ± 2.25
Total dose administered (µg)	102.2 ± 34.56	118.7 ± 35.05	110.6 ± 37.71	104.0 ± 35.01
Prescribed daily starting dose based on approved algorithm^a^	779 (94.2)	81 (96.4)	32 (97.0)	892 (94.5)
Dose deviations				
Prescribed daily starting dose not based on the approved algorithm^a^	48 (5.8)	3 (3.6)	1 (3.0)	52 (5.5)
***Dose deviations from dose calculated using approved algorithm and prescribed daily starting dose (µg)***				
n	814	84	33	931
Dose deviation between calculated and prescribed dose	0.21 ± 1.42	0.1 ± 0.88	0.6 ± 1.90	0.2 ± 1.40
Higher dose (>0.33 µg)*^a^*	60 (7.3)	2 (2.4)	4 (12.1)	66 (7.0)
Nearly the same dose ( ± 0.33 µg)*^a^*	715 (86.5)	79 (94.0)	28 (84.8)	822 (87.1)
Lower dose (> –0.33 µg)*^a^*	39 (4.7)	3 (3.6)	1 (3.0)	43 (4.6)
Daily dose adjusted during ovarian stimulation^a^	44 (5.3)	7 (8.3)	6 (18.2)	57 (6.0)
If daily dose adjusted, type of adjustment^b,c^				
Increased	31 (3.7)	3 (3.6)	4 (12.1)	38 (4.0)
Decreased	13 (1.6)	6 (7.4)	3 (9.1)	22 (2.3)

Data are mean ± SD or n (%).

^a^Percentages calculated using total number of women in study (N).

^b^Percentages calculated from patients with daily dose adjusted (n).

^c^Percentages sum to >100% because women could be included in more than one category.

GnRH, gonadotrophin-releasing hormone; n, number of patients in specific category; N, number of patients; SD, standard deviation.

#### 3.2.2 Use of approved dosing algorithm for calculating starting dose for Cycle 1

Nearly all women (893/944 [94.5%]) were prescribed follitropin delta after their physician had calculated the starting dose according to the dosing algorithm, although some physicians then adjusted the prescribed starting dose. The mean difference between the daily starting dose of follitropin delta prescribed by the treating physician and the dose calculated by the per-label algorithm was small ( ± 0.21 µg, including women with no starting dose deviation). Most women (822/944, 87.1%) were prescribed follitropin delta within 0.33 µg (1 click of pen) of the dose calculated using the approved dosing algorithm.

#### 3.2.3 Deviations from per-label dosing during Cycle 1

Three main types of deviations were observed: **1**) prescribed dose was different from the calculated starting daily dose (i.e., starting dose deviations), reported for 165 women (17.5%), with half of these within 0.66 µg (1 or 2 clicks of pen, n=86 [9.1%]) and one-third within 0.33 µg (1 click of pen, n=59 [6.3%]); **2)** daily dose changes during stimulation (i.e., dose adjustments), reported for 57 (6.0%) women (38 women received dose increases and 22 received dose decreases during their follitropin delta dosing period); and **3)** addition of another gonadotropin during OS (i.e., regimen deviation: change from monotherapy with follitropin delta to a mixed FSH regimen), reported for 33 women (3.5%). Women who received follitropin delta as part of a mixed FSH regimen were more likely to have a starting dose deviation or dose adjustments during stimulation. As so few women received a mixed FSH therapy regimen, we reported ovarian response and pregnancy outcomes for the whole subgroup but not separately according to GnRH protocol.

Reasons for starting dose deviations included physicians prescribing lower follitropin delta doses to avoid OHSS (based on the physician’s clinical experience) or higher doses to achieve a better ovarian response for women with very low baseline AMH levels. Similarly, reasons for dose increases during stimulation were normally due to insufficient initial ovarian response (e.g., low antral follicle count at Day 3 of cycle) and dose decreases were made due to potential high ovarian responses (e.g., elevated estradiol levels or high antral follicle count at Day 3 of cycle). A few women were reported to have dose adjustments due to changes to bodyweight (n=2) or had updated AMH levels reported after they had started their cycle (n=3). Reasons for prescribing follitropin delta as part of a mixed FSH were not reported.

#### 3.2.4 Dosing according to GnRH protocol use during Cycle 1

**Figure 3:**
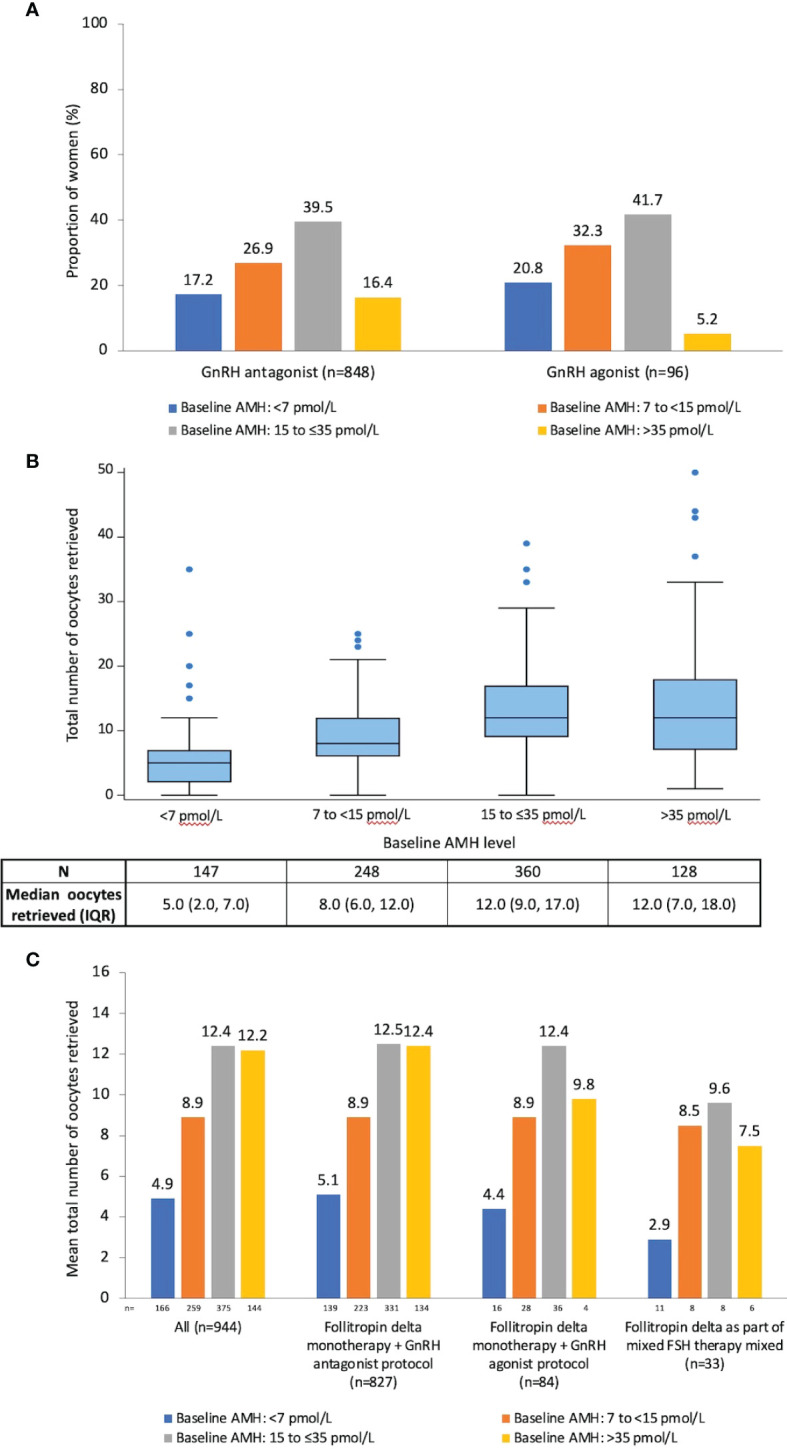
Baseline AMH subgroups and ovarian response for Cycle 1. **(A)** Women grouped by baseline AMH subgroup and GnRH protocol (n=944);**(B)** Distribution of the total number of retrieved oocytes according to baseline AMH subgroup for all women who underwent oocyte retrieval (n=885). For each category, the box defines the 25th and 75th percentiles of the distribution (i.e., the interquartile range), with the line inside the box representing the median. The whiskers represent the 5th and 95th percentiles of the distribution. Values outside the whisker boundaries are represented as individual dots; **(C)** Mean number of retrieved oocytes by AMH and GnRH protocols for all women who initiated Cycle 1 (n=944). AMH, anti-Müllerian hormone; GnRH, gonadotropin-releasing hormone; IQR, interquartile range.

A total of 848 women (89.8%) received a GnRH antagonist protocol and 96 (10.2%) received a GnRH agonist protocol; the mean total dose of follitropin delta (for monotherapy and mixed FSH regimens) was 102.5 µg and 116.8 µg for the antagonist and agonist groups, respectively. For women who received follitropin delta as monotherapy in a GnRH antagonist protocol, their mean total dose was 102.2 µg (mean daily starting dose 10.1 µg; n=827). For women who received follitropin delta as monotherapy in a GnRH agonist protocol, their mean total dose was 118.7 µg (mean daily starting dose 10.8 µg; n=84). The median duration of treatment with follitropin delta was 10 days for women using an antagonist protocol and 11 days for women using an agonist protocol. A slightly larger proportion of women who received an agonist protocol had a baseline AMH of <7 pmol/L (<0.98 ng/mL) compared with those who received an antagonist protocol (20.8% and 17.2%, respectively; **Figure 3A**).

#### 3.2.5 Dosing according to IVF/ICSI, triggering and fresh/frozen transfer procedures for Cycle 1

There was no dosing difference for follitropin delta according to different protocols used for trigger of final follicular maturation, type of transfer procedure or luteal phase support during Cycle 1 (data not shown).

### 3.3 IVF/ICSI and triggering procedures for Cycle 1

Among women with available data (n=879), the most frequent fertilization technique was ICSI, which was performed for 577 women (61.1%). IVF was used for 216 women (22.9%) and the remaining 86 women (9.1%) received both IVF and ICSI. Triggering of final follicular maturation at Cycle 1 was carried out for 893 women (**Table S1**).

### 3.4 Ovarian response for Cycle 1

A total of 885/944 women (93.8%) underwent oocyte pick-up. The mean number of retrieved oocytes per woman in Cycle 1 was 10.1. Women with baseline AMH <7 pmol/L were more likely to have a lower ovarian response (**Figure 3B**). Follitropin delta dose deviations versus no dose deviations (data not shown) and the use of GnRH antagonist versus GnRH agonist were not associated with differences in ovarian response (**Figure 3C**). An acceptable ovarian response (4–19 retrieved oocytes) was attained by 702/944 women (74.4%). The mean number of retrieved oocytes decreased by increasing age category, ranging from 11.8 ± 7.64 oocytes retrieved, for women <35 years old to 5.3 ± 4.66 retrieved oocytes for women >40 years old.

### 3.5 Transfer procedures for Cycle 1

A total of 775/944 women (82.1%) received at least one embryo/blastocyst transfer (fresh and/or frozen; mean number of transfers: 1.6 ± 0.96, range 1–5), without clear differences between GnRH antagonist and agonist protocols. Approximately two-thirds of women (62.7% [n=592]) had a fresh transfer, just over one-third (39.5% [n=371]) had at least one frozen transfer and nearly one-fifth (17.9% n=169) had no transfer. There were 221 women who had all their embryos/blastocysts frozen, with the most frequently reported reasons being either the clinic policy and pre-agreed treatment plan, related to the COVID-19 pandemic or the women’s unfavorable progesterone levels. During Cycle 1 (fresh and/or frozen), 666 women received luteal phase support, the majority of whom received progesterone (n=579).

### 3.6 Pregnancy outcomes for Cycle 1

Pregnancy outcomes by GnRH protocol for Cycle 1 are presented in **Table 3**. After a fresh transfer, 352 women were reported to have a positive βhCG test (37.3% [352/944]). Clinical and vital pregnancies at 5–6 weeks were reported for 290 (30.7%) and 279 (29.6%) women, respectively. There was a trend for rates of positive βhCG, clinical pregnancy and vital pregnancy to be slightly lower for the women with baseline serum AMH <7 pmol/L regardless of which GnRH protocol was used (data not shown). The ongoing pregnancy rate was 27.0% (n=255) for those who initiated Cycle 1, and 43.1% per fresh transfer. Cumulative ongoing pregnancy rate was 36.4% (n=344) per initiated cycle, and 44.4% per transfer. Fifteen women reported pregnancy loss (spontaneous abortion, n=13; elective abortion, n=2).

**Table 3:** Pregnancy outcomes by GnRH protocol for Cycle 1.

	Follitropin delta as FSH monotherapy		Follitropin delta as part of combination FSH therapy (N=33)	Overall(N=944)	
	GnRH antagonist(N=827)	GnRH agonist(N=84)		
**Patients with fresh transfer, n (%)^a^**	515 (62.3)	60 (71.4)	17 (51.5)		592 (62.7)
Positive βhCG test, n (%)^a^	317 (38.3)	25 (29.8)	10 (30.3)		352 (37.3)
Clinical pregnancy, n (%)^a,b^	258 (31.2)	22 (26.2)	10 (30.3)		290 (30.7)
Vital pregnancy^c^	250 (30.2)	*21 (25.0)*	8 (24.2)		279 (29.6)
Ongoing pregnancy, n (%)^a,d^	229 (27.7)	19 (22.6)	7 (21.2)		255 (27.0)
Pending ongoing pregancy result	5 (0.6)	1 (1.2)	1 (3.0)		7 (0.7)
Pregnancy rate per fresh transfer^f^	44.5%	31.7%	41.2%		43.1%
Implantation rate per started cycle, %^e^	34%	30%	30%		33%
**Patients with at least one transfer (fresh and/or frozen transfer), n (%)^a^**	683 (82.6)	67 (79.8)	25 (75.6)		775 (82.1)
Cumulative ongoing pregnancy, n (%)^a,g^	310 (37.5)	26 (31.0)	8 (24.2)		344 (36.4)
Pending ongoing pregnancy result	9 (1.1)	1 (1.2)	1 (3.0)		11 (1.2)
Pregnancy rate per transfer^f^	45.4%	38.8%	32.0%		44.4%

^a^Percentages calculated using total number of women in study (N).

^b^Clinical pregnancy, defined as at least 1 gestational sac 5–6 weeks after transfer.

^c^Vital pregnancy, defined as at least 1 intrauterine gestational sac with fetal heartbeat 5–6 weeks after transfer.

^d^Ongoing pregnancy, defined as at least 1 intrauterine viable fetus 10–11 weeks after transfer.

^e^Implantation rate, defined as the proportion of transferred embryos/blastocysts that resulted in intrauterine viable fetuses at 10-11 weeks after transfer. This only includes women with fresh transfer. Some women had multiple embryos/blastocysts transferred.

^f^Pregnancy rates per transfer were calculated using the number of ongoing pregnancies per number of embryo/blastocyst transfers.

^g^Cumulative ongoing pregnancy includes the number of women with at least one ongoing pregnancy following a fresh transfer and/or any frozen transfer using embryos from Cycle 1.

βhCG, beta human chorionic gonadotropin; FSH, follicle stimulating hormone; GnRH, gonadotrophin-releasing hormone.

### 3.7 Cycle cancellation for Cycle 1

Most women (n=889 [81.3%]) successfully completed Cycle 1 with follitropin delta (**Table S2**). A total of 5.8% of women (n=55) had their cycle cancelled prior to oocyte collection and 12.9% women (n=122) had their cycle cancelled after oocyte collection. The most common reason for cancellation prior to oocyte collection was poor ovarian response (n=32; 3.5%), followed by excessive ovarian response (n=3; 0.3%). The most common reason for cancellation after oocyte collection was ‘Other’ reason (n=41; 4.3%), for whom 17 women had the reason listed as to prevent or avoid OHSS and the rest were mostly related to clinical complications, such additional embryonic screening for genetic mutation(s), hydrosalpinx, post-pick-up bleeding, spontaneous pregnancy, no semen available, or endometrial polyp. Eleven women (1.2%) had cycles cancelled after pickup due to OHSS.

### 3.8 Interventions used to prevent potential early OHSS during Cycle 1

All treatment decisions for preventing potential early OHSS were according to local practices. Overall, 156 women (16.5%) received preventive interventions for potential early OHSS (before Day 9 after triggering). Preventive interventions were administered to 142/827 women (17.2%) who had received a monotherapy antagonist protocol; 7/84 (8.3%) who had received a monotherapy agonist protocol; 4/21 (19.0%) who had received a mixed antagonist protocol and 3/12 (25.0%) who had received a mixed agonist protocol. Overall, preventive interventions for potential early OHSS used during Cycle 1 were coasting (n=8), triggering of final follicular maturation with GnRH agonist with fresh transfer (n=12), triggering of final follicular maturation with GnRH agonist with a freeze-all strategy (n=107), dopamine agonist (n=10), colloid infusion (n=4), plasma expander (n=1) and the remaining 27 women mostly received a combination of the previous interventions. Women who required a dopamine agonist, colloid infusion or plasma expander were likely to have had early OHSS which needed treatment, whereas the decision to use a freeze-all strategy may have been due to a combination of clinical factors, not just to prevent or avoid OHSS (see reasons for cycle cancellation above). The most frequently used preventive intervention for women who had received a follitropin delta monotherapy plus antagonist protocol was triggering of final follicular maturation with GnRH agonist with a freeze-all strategy (n=105/827 [12.7%]). Of the seven women who had received a follitropin delta monotherapy plus GnRH agonist protocol and who had received preventive intervention for potential early OHSS, six were confirmed not to have received any agonist trigger (one woman had missing data). There were 10 cycle cancellations among the 156 women who received preventive interventions for potential early OHSS.

### 3.9 Safety for all cycles

For the safety analysis, 944 women underwent a total of 1130 OS cycles. ADRs are summarized in **Table 4**. Overall, 49 women (5.2%) reported 58 ADRs, with four women experiencing six ADRs leading to treatment and study discontinuation (OHSS, n=2 events; vomiting, n=1 event; headache, n=1 event; rash, n=1 event and premature ovulation, n=1 event). Most ADRs (51/58 [87.9%]) were mild or moderate in intensity. During the whole study period the occurrence of ADRs was low, with most types of ADR occurring in one or two women (≤0.2%), except for OHSS. No deaths were reported during the study.

**Table 4:** Adverse drug reactions.

	Participating women (N=944)	
	N (%)^a^	Number of events
Women with at least one ADR	49 (5.2)	58
Women with at least one ADR leading to treatment withdrawal	4 (0.4)	6
Women with at least one ADR with severe intensity^b^	7 (0.7)	7
Women with at least one serious ADR^b^	12 (1.3)	12
Women with at least one serious ADR leading to treatment withdrawal	0	0
Women with at least one serious ADR with severe intensity^b^	5 (0.5)	5
Women with at least one serious ADR leading to death	0	0
All ADRs (any severity)		
Ovarian hyperstimulation syndrome	37 (3.9)	37
Headache	2 (0.2)	2
Mood altered	2 (0.2)	2
Fatigue	2 (0.2)	2
Vomiting	2 (0.2)	2
Affect lability	1 (0.1)	1
Anxiety	1 (0.1)	1
Diarrhea	1 (0.1)	1
Dry skin	1 (0.1)	1
Endometriosis	1 (0.1)	1
High response to ovarian stimulation	1 (0.1)	1
Mood swings	1 (0.1)	1
Nausea	1 (0.1)	1
Premature ovulation	1 (0.1)	1
Pruritus	1 (0.1)	1
Rash	2 (0.2)	2
Swelling	1 (0.1)	1

^a^Percentages calculated using total number of women in study (N).

^b^All reported ADRs with severe intensity and all reported serious ADRs were cases of OHSS, and nine of these women with OHSS were admitted to hospital. See text for more details.

ADR, adverse drug reaction; OHSS, ovarian hyperstimulation syndrome.

#### 3.9.1 OHSS occurrence during all cycles

The most frequently reported ADR was OHSS (n=37 [3.9%]), which lasted a median of 9 days; most cases of OHSS were of mild or moderate intensity (n=30 [3.2%]). After triggering of follicular maturation, OHSS presentation was a median of 6 days for 32 women who had timing of OHSS reported, with 20 OHSS events (54.1%) occurring at or before 9 days after triggering, and 12 events (32.4%) occurring after 9 days. Overall, seven women (0.7%) had OHSS of severe intensity, which lasted a median duration of 9 days, three of which occurred ≤9 days after triggering of follicular maturation and three occurred after 9 days (timing was missing for the seventh woman with severe OHSS). Nine women were hospitalized with OHSS, for a median duration of 6.0 days. In total, 16 of the 37 women who developed OHSS had received preventive treatment. All 37 women recovered from their OHSS episode without sequalae.

## 4 Discussion

The PROFILE study is the first real-world multinational observational study to explore utilization patterns, effectiveness and safety of follitropin delta in daily clinical practice. Follitropin delta is the first and only FSH used for OS that uses an individualized fixed daily dose based on the woman’s bodyweight and AMH levels. Most of the participants received follitropin delta as monotherapy without starting dose deviations or dose adjustments (i.e., according to the approved label). Physicians used the follitropin delta dosing algorithm for 95% of participants, although some made minor adjustments to the prescribed starting dose or adjusted the dose during the OS cycle based on clinical factors. In PROFILE, the mean total dose of follitropin delta (104 µg) was slightly higher than observed in randomized clinical trials, in which the mean total dose was 90.0 µg, 83.5 µg and 77.5 µg for the ESTHER-1, STORK and GRAPE trials, respectively (11–13). The higher total dose observed in PROFILE can be explained by the differences in duration of stimulation (~9 days in ESTHER-1, GRAPE and STORK compared with 10 days in PROFILE) as well as differences in participating women’s bodyweight (mean bodyweight was ~10 kg lower for women participating in GRAPE and STORK, and 2.4 kg lower for women participating in ESTHER-1 compared with PROFILE) and baseline AMH levels (median AMH concentrations were higher for women in the GRAPE [23.4 pmol/kg] and STORK [18.2 pmol/kg] trials compared with PROFILE [16.4 pmol/kg]). Moreover, the ESTHER-1, GRAPE and STORK trials only included women with a BMI between 17.5 and 32 kg/m^2^ whereas PROFILE had no BMI limit, allowing obese women to enroll. Regardless of the differences in follitropin delta utilization among these studies, PROFILE demonstrates that the follitropin delta dosing algorithm based on bodyweight and AMH levels allows for acceptable ovarian responses and ongoing pregnancy rates that were similar to, or higher than, the rates observed in RCTs with follitropin delta (11, 13, 18, 23). PROFILE is in line with the findings of a recent retrospective study of 360 women undergoing ovarian stimulation with follitropin delta for IVF/ICSI at eight German fertility clinics (24).

Nearly all women who received either starting dose deviations or dose modifications during Cycle 1 were prescribed a daily dose of follitropin delta within 0.33 µg or 0.66 µg of the algorithm-calculated dose (1 or 2 clicks of the injection pen) and importantly, women with these small deviations in follitropin delta dosing showed no discernable difference in ovarian response compared with those who received follitropin delta as per the approved label regimen. The ovarian response, analyzed by AMH subgroup, confirms that the dosing algorithm for follitropin delta is effective at producing a predictable ovarian response in real-world clinical settings, regardless of the type of GnRH protocol used. Moreover, most physicians did not make follitropin delta dose modifications during stimulation.

As expected, women with AMH <7 pmol/L had fewer oocytes retrieved indicating that ovarian reserve is an important factor for predicting the number of retrieved oocytes. Previous studies show that higher doses of FSH for women with low ovarian reserve do not result higher live birth rates (25, 26).

A total of 43% women who underwent a fresh transfer had an ongoing pregnancy at Cycle 1 (27% of women who initiated Cycle 1), and the cumulative ongoing pregnancy rate was 36.4% for initiated cycle. This demonstrates that OS with an individualized follitropin delta dosing regimen results in a high rate of pregnancies in real-world clinical practice, supporting pregnancy rates observed in RCTs (11–14). There were no identified signals for reported pregnancy loss.

As PROFILE was a non-interventional study, investigators treated women at risk of OHSS as they would normally do according to local clinical practice. Although national guidelines are generally similar, there are subtle differences in OHSS severity classification and treatment guidance, including when hospitalization is necessary (27–29). As such, country-specific guidelines should be taken into consideration if comparisons are made between PROFILE and other trials with regards to the treatment of women with OHSS. When considering all cases of OHSS, a similar proportion of women experienced any OHSS in PROFILE compared with RCTs for follitropin delta, ESTHER-1 (3.9% in PROFILE and 3.5% in the first cycle of ESTHER-1); however, there was greater use of preventive interventions for potential early OHSS among women enrolled in the PROFILE study compared with the ESTHER-1 trial (16.5% in PROFILE and 2.3% in ESTHER-1) (11). This was possibly due the stricter use of *per protocol* criteria for preventive interventions for OHSS in the ESTHER-1 trial and reflects a more cautious approach among physicians when it comes to prescribing preventive interventions for potential early OHSS in clinical practice. In addition, the PROFILE study’s inclusion/exclusion criteria allowed enrolment of women with a broad range of comorbid health conditions, including polycystic ovary syndrome and metabolic disorders, representing a wide range of women requiring fertility treatment with confounding factors for OHSS risk that were not considered for this study. In both ESTHER-1 and 2, follitropin delta resulted in a lower incidence of any OHSS than the comparator, follitropin alfa (3.5% vs 4.8%) (17).

In PROFILE, most patients (~90%) received a GnRH suppression protocol with an GnRH antagonist, in line with the evidence from RCTs for follitropin delta (11, 12, 17). Although a relatively small proportion of women (~10% of the study population) received a GnRH agonist protocol, our results from the PROFILE study are one of the first reports of real-world use of combining follitropin delta with an agonist protocol. Among women who received an agonist protocol, a slightly higher proportion had a baseline AMH of <7 pmol/L (<0.98 ng/mL) compared with those who received an antagonist protocol. We observed a similar mean number of oocytes collected for women who underwent an agonist protocol as for those who had an antagonist protocol, which may be due to the lower ovarian reserve among women who received an agonist protocol. Normally, women who receive agonist protocols potentially have more oocytes to be retrieved compared with antagonist protocols (30). Long GnRH agonist protocols are generally associated with one more oocyte retrieved (vs antagonist protocols), one extra day of stimulation and a higher total dose of exogenous FSH (that is equivalent to the extra day of dosing) (31, 32). Our study aligns with the dosing regimens of follitropin delta when used with an agonist protocol, but not the one extra oocyte retrieved. In addition, fewer women who received an agonist protocol received a preventive intervention for potential early OHSS than those who received an antagonist protocol, which again could have been because these women were more likely to have a low ovarian response and therefore at lower risk of OHSS. Generally, long GnRH agonist protocols are associated with a higher risk of OHSS than antagonist protocols (33). A recent RCT of follitropin delta used with a long GnRH agonist protocol showed that the women had a mean of 12.5 oocytes retrieved and an ongoing pregnancy rate of 43% (n=104) per started cycle, supporting our results that follitropin delta is effective when used with a long GnRH agonist protocol (23). Another RCT is currently ongoing to compare the use of follitropin delta in GnRH antagonist versus agonist protocols (BEYOND, NCT03809429).

### 4.1 Study strengths and limitations

The main strengths of this study are its large size, heterogenous population representing the broad range of patients seen in reproductive health clinics and its prospective design. PROFILE comprised 944 women undergoing OS for IVF/ICSI fertility treatment with a broad range of characteristics who followed routine clinical practice in multiple countries and clinics; however, enrolment was not balanced across countries meaning that there may be a bias towards treatment protocols used in countries with the highest enrolment. For women with OHSS, severity grade was not recorded due to differences in local practice guidelines for treating and grading OHSS. As such, we do not know how many women would have been graded as having severe OHSS.

We also did not anticipate physicians prescribing a mixed FSH regimen during the study. Although only a small proportion of participants received a mixed FSH regimen, the reasons for prescribing follitropin delta as part of a mixed FSH regimen were not recorded, nor were the doses of the other administered FSH preparations used for OS.

Another potential limitation of our study is that we did not record live birth rates or neonatal outcomes. Although these outcomes are now part of the core outcomes for infertility research (34), enrollment for the PROFILE study started before these outcomes had been published. Nonetheless, live birth rates after ovarian stimulation with follitropin delta have previously been established in Phase 3 studies and were almost the same as ongoing pregnancy rates in these trials (11–13). Pregnancy and neonatal outcomes after OS with follitropin delta have also previously been reported (14). For this observational study, the ongoing pregnancy rate was deemed sufficient to confirm the effectiveness of follitropin delta when compared to previously published RCTs.

Although we had originally planned to observe up to three consecutive OS cycles with follitropin delta, the COVID-19 pandemic led to the temporary closure of fertility clinics and we made the decision to terminate the study early once we had enough participants for the primary outcome analysis; however, this meant that many participants could not start subsequent OS cycles. As such, too few participants started Cycles 2 and 3 to allow meaningful analysis of these data.

Real-world observational studies, such as PROFILE, complement data from RCTs, and provide reassurance about effectiveness and safety in a broader range of women compared to the strict inclusion/exclusion criteria often necessary for RCTs to prevent confounding variables. Although clinical decisions are normally based on data from gold standard RCTs, evidence of real-world effectiveness is particularly important for policy makers and payers when deciding access to treatments. Nonetheless, there are a few publications that discuss the pharmacoeconomic impact of follitropins and most compare follitropin alfa to its biosimilars (35–37). As such, further research is ongoing to assess the overall cost effectiveness of follitropin delta when used for OS as part of IVF/ICSI therapy, including any potential cost savings in reduced rates of OHSS in the first OS cycle, compared with other follitropins.

### 4.2 Overall conclusions

This first large real-world study among a broad range of women naïve to OS supports the efficacy and safety profile of follitropin delta previously demonstrated in randomized controlled trials (11–13, 17). In PROFILE, nearly all patients (95%) had their starting dose calculated using the approved algorithm and most women (87%) received follitropin delta within 0.33 µg (one pen click) of the algorithm-recommended dose. Most women received a GnRH antagonist protocol. Real-world use of individualized dosing of follitropin delta is effective with 74.4% of women attaining 4–19 retrieved oocytes in their first OS cycle. During the first cycle, the ongoing pregnancy rate per fresh transfer was 43%, confirming the efficacy of follitropin delta demonstrated in pivotal clinical trials (11–13). Discontinuation of follitropin delta was rare, with only four women stopping the drug before the end of their dosing regimen. No new safety signals for follitropin delta were reported, and OHSS incidence was within the expected range for the broad range of women undergoing their first OS cycle.

## Data availability statement

The original contributions presented in the study are included in the article/**Supplementary Materials**. Further inquiries can be directed to the corresponding author.

## Ethics statement

The studies involving human participants were reviewed and approved by Melbourne IVF Human Research Ethics Committee, Australia; Bellberry Human Research Ethics Committee, Australia; IVFAustralia Ethics Committee, Australia; Ethikkommission der Medizinischen Universität Innsbruck, Austria; Commissie Medische Ethiek, Vrije Universiteit Brussels, Belgium; Cliques universitaires de Bruxelles, Hopital Erasme, Belgium; Universitat zu Lubeck ethics committee, Germany; Veritas Review Board, Quebec, Canada; Single Regional Ethics Committee, Scientific Institute for Research, Hospitalization and Healthcare, *Via* Franco Gallini, Italy; Comitato Etico Lazio 2, Rome, Italy; Comitato Etico Regionale per la Sperimentazione Clinica della Regione Toscana, Italy; Comitato Etico di Area Vasta Sud Est, Toscana, Italy; Il Comitato Etico dell’Ospedale San Raffaele - Milano Istituto di Ricovero e Cura a Carattere Scientifico, Milan, Italy; Medische Ethische Toetsings Commissie, Erasmus MC Universitair Medisch Centrum Rotterdam, Netherlands; Diakonessenhuis Utrecht - Zeist - Doorn, Netherlands; Komisja Bioetyczna, Kliniki iLaboratoria Medyczne Invicta, Rajska, Gdansk, Poland; Fundacio Puigvert ethics committee, Barcelona, Spain; IVIRMA, Barcelona, Spain; and NHS Health Research Authority London- Surry Research Ethics committee, United Kingdom. The patients/participants provided their written informed consent to participate in this study.

## Author contributions

CB: study conception, study design, data acquisition, data analysis, drafting and critically reviewing multiple versions of the manuscript, and approval of the final version. GG: study conception, study design, data acquisition, data analysis, drafting and critically reviewing multiple versions of the manuscript, and approval of the final version. RR: data acquisition, critically reviewing multiple versions of the manuscript, and approval of final version. PL: study design, data analysis, statistical analysis plan, drafting and critically reviewing multiple versions of the manuscript, and approval of final version. YLYS: data analysis, drafting and reviewing multiple versions of the manuscript, and approval of final version. SR: study design, data analysis, statistical analysis plan, draft and critically reviewing multiple versions of the manuscript, and approval of final version. JSEL: data acquisition, drafting and critically reviewing multiple versions of the manuscript, and approval of the final version. All authors contributed to the article and approved the submitted version.
