# Supplementary material for: Prospective multicenter non-interventional real-world study to assess the patterns of use, effectiveness and safety of follitropin delta in routine clinical practice (the PROFILE study)
Source: Front Endocrinol (Lausanne). 2022 Dec 22;13:992677. doi: 10.3389/fendo.2022.992677 (PMC9815701; doi:10.3389/fendo.2022.992677)
Supplement: Supplementary file 1 [file Table_1.docx]

## Table S1. Triggering of final follicular maturation at Cycle 1

|  | **Participating women (N=944)** |
| --- | --- |
| **Patients with triggering, n^a^** | **893** |
| GnRH agonist | 91 |
| GnRH antagonist | 802 |
| **Patients with hCG trigger, n** | **764** |
| GnRH agonist | 91 |
| GnRH antagonist | 673 |
| **Patients with GnRH agonist trigger, n** | **159** |
| **Patients with dual trigger, n^b^** | **30** |
| **Patients without triggering, n^c^** | **50** |
| GnRH agonist | 5 |
| GnRH antagonist | 45 |
| **Patients with missing records for triggering, n** | **1** |

^a^ Patients with hCG and/or GnRH administered.
^b^ Patients with hCG and GnRH administered.
^c^ Patients without hCG nor GnRH administered.

GnRH: gonadotropin-releasing hormone; hCG: human chorionic gonadotropin; n: number of patients in specific category.
