# Supplementary material for: Prospective multicenter non-interventional real-world study to assess the patterns of use, effectiveness and safety of follitropin delta in routine clinical practice (the PROFILE study)
Source: Front Endocrinol (Lausanne). 2022 Dec 22;13:992677. doi: 10.3389/fendo.2022.992677 (PMC9815701; doi:10.3389/fendo.2022.992677)
Supplement: Supplementary file 2 [file Table_2.docx]

## Table S2. Reasons for Cycle 1 cancellation

|  | **Participating women (N=944)** |
| --- | --- |
| **Cancellation before oocytes pickup, n (%)^a^** |  |
| No | 889 (94.2) |
| Yes | 55 (5.8) |
| **If yes, reason for cancellation before oocytes pick up, n (%)** |  |
| Poor ovarian response | 32 (3.4) |
| Excessive ovarian response | 3 (0.3) |
| Patient not taking triggering method at the correct time | 2 (0.2) |
| Patient choice | 3 (0.3) |
| Any illness which prevents oocyte collection procedure | 1 (0.1) |
| Other | 14 (1.5) |
| **Cancellation after oocytes pickup, n (%)^a^** |  |
| No | 822 (87.1) |
| Yes | 122 (12.9) |
| **If yes, reason for cancellation after oocytes pickup, n (%)** |  |
| No oocytes collected | 8 (0.8) |
| No oocytes fertilized | 24 (2.5) |
| Abnormal fertilization | 5 (0.5) |
| Abnormal embryo development | 8 (0.8) |
| No embryo development | 23 (2.4) |
| OHSS | 11 (1.2) |
| Patient choice | 2 (0.2) |
| Other | 41 (4.3) |

^a^ Percentages calculated from women included in Cycle 1 (N).

OHSS: ovarian hyperstimulation syndrome.
